# Classification of Prolapsed Mitral Valve versus Healthy Heart from Phonocardiograms by Multifractal Analysis

**DOI:** 10.1155/2013/376152

**Published:** 2013-05-20

**Authors:** Ana Gavrovska, Goran Zajić, Irini Reljin, Branimir Reljin

**Affiliations:** ^1^Research and Development Department, Innovation Center of the School of Electrical Engineering in Belgrade, Bulevar Kralja Aleksandra 73, 11120 Belgrade, Serbia; ^2^Department of Telecommunications and Information Technology, School of Electrical Engineering, University of Belgrade, Bulevar Kralja Aleksandra 73, 11120 Belgrade, Serbia; ^3^Department of Telecommunications, ICT College of Vocational Studies, Zdravka Čelara 16, 11000 Belgrade, Serbia

## Abstract

Phonocardiography has shown a great potential for developing low-cost computer-aided diagnosis systems for cardiovascular monitoring. So far, most of the work reported regarding cardiosignal analysis using multifractals is oriented towards heartbeat dynamics. This paper represents a step towards automatic detection of one of the most common pathological syndromes, so-called mitral valve prolapse (MVP), using phonocardiograms and multifractal analysis. Subtle features characteristic for MVP in phonocardiograms may be difficult to detect. The approach for revealing such features should be locally based rather than globally based. Nevertheless, if their appearances are specific and frequent, they can affect a multifractal spectrum. This has been the case in our experiment with the click syndrome. Totally, 117 pediatric phonocardiographic recordings (PCGs), 8 seconds long each, obtained from 117 patients were used for PMV automatic detection. We propose a two-step algorithm to distinguish PCGs that belong to children with healthy hearts and children with prolapsed mitral valves (PMVs). Obtained results show high accuracy of the method. We achieved 96.91% accuracy on the dataset (97 recordings). Additionally, 90% accuracy is achieved for the evaluation dataset (20 recordings). Content of the datasets is confirmed by the echocardiographic screening.

## 1. Introduction

Cost effectiveness in cardiovascular monitoring represents one of the great challenges [1]. Besides traditional auscultation, phonocardiography has become a valuable diagnostic tool. It has shown a great potential for developing low-cost computer-aided diagnosis (CAD) systems [2–4]. Efficient phonocardiography analysis can decrease the necessity for more complex methods.

Phonocardiograms (PCGs) represent digital records of heart sounds that are believed to yield valuable clinical information. One of the major concerns regarding phonocardiograms is recognizing and understanding such relevant information. So far, very little work has been reported on automatic detection of relevant clinical information and diagnosis through phonocardiography.

This paper represents a step towards automatic detection of one of the most common pathological syndromes, so-called mitral valve prolapse (MVP), using phonocardiograms and multifractal analysis. Even though the examination of existing pathological syndromes is possible by observing and listening to the PCGs, it is believed that automatic detection of a possible abnormality is of great importance. It is also important to draw attention of a physician or an examiner to such cardiac events.

Characteristic midsystolic click with possible late systolic murmur represents a typical auscultatory finding when MVP is diagnosed [5–10]. In phonocardiography, this property is described in terms of the pathological functionality of mitral valve leaflets. Time domain visual analysis of such clicks is a demanding and an error-prone process. The process of examination of each beat separately within a PCG record is also time consuming.

The dynamics of nonstationary medical signals, such as cardiosignals, may be analyzed by different signal processing tools, where fractals, multifractals, and wavelets are of particular interest [11–16]. The nature of self-similarity enables finding features on one scale which resemble the features from another scale, despite the complexity of the analyzed object or system dynamics.

So far, the multifractal analysis shows to be suitable for investigating heartbeat dynamics [15]. Multifractal behaviour of the cardiovascular system is investigated through analysis of Hölder exponents and fractal behavior using different approaches, such as detrended fluctuation analysis (DFA) and self-affine fractal variability analysis of heartbeat dynamics [17, 18], local variability analysis [19, 20], analysis of healthy and pathological dynamics from the aspect of respiratory rate variability [21] and brain activity in healthy patients [21, 22], analysis of Hölder exponents using laser Doppler flowmetry technique [23], analysis of progressive central hypovolemia influence [24, 25], estimation of heart rate variability (HRV) large deviations [26], and arrhythmia [27]. Many variations of cardiosignals are examined using multifractality, age-related changes [28, 29], as well as changes in heart dynamics: before and after particular treatment [30], for different human races [31], body positions [23], and during wake and sleep phases [32, 33]. Heartbeat dynamics is compared between a group of healthy patients and a group of patients with heart failures [17]. The records of the heart failure group show a loss of multifractality and narrowing of the multifractal spectra. Long-range multiscale properties are investigated for congestive heart failure (CHF) disease and primary autonomic failure (PAF) disease. Differences in singularity spectra of the heart rate signals are shown for healthy, CHF, and PAF group [34].

Very little research is reported in the literature regarding phonocardio diagnostics from the multifractal viewpoint. Multifractality in heartbeat dynamics can be considered through acoustic heartbeats. Both global and local (interbeat) techniques are introduced in heartbeat dynamics for detection of abnormalities [35]. Although the global-based concept, such as Legendre multifractal spectrum, seems to be blind to subtle features in case of large heart rate deviations [26], we show in this paper that this is not the case with detection of PCG records of patients with PMV in a dataset consisting of records from both healthy patients and patients with PMV.

We propose a novel approach for distinguishing PCGs that belong to patients with PMVs and healthy patients, based on a multifractal analysis. Multifractal analysis is used in a global manner (without click event segmentation in the time domain).

The paper is organized as follows. In Section 2, the dataset acquisition procedure is shortly explained.  Section 3 gives the fundamental background of PMV and phonocardiogram morphology in patients with PMV. Furthermore, it provides an introduction to the fundamentals of multifractals and their suitability for medical signal analysis. In Section 4, we propose an algorithm for distinguishing records with PMV and healthy records using phonocardiography. Obtained results are presented in Section 5. Finally, we give conclusions based on the proposed algorithm.

## 2. Data Acquisition

In the study, 117 children (7–19 years old) contributed to acquisition of 117 recordings. There were 54 male and 63 female patients (*M* = 54, *F* = 63). No multiple-day recordings were made. From this dataset, 97 recordings (*M* = 45, *F* = 52) are used for setting of the parameters of the classifier. The rest of the recordings are included in the validation study.

**Figure 1:**
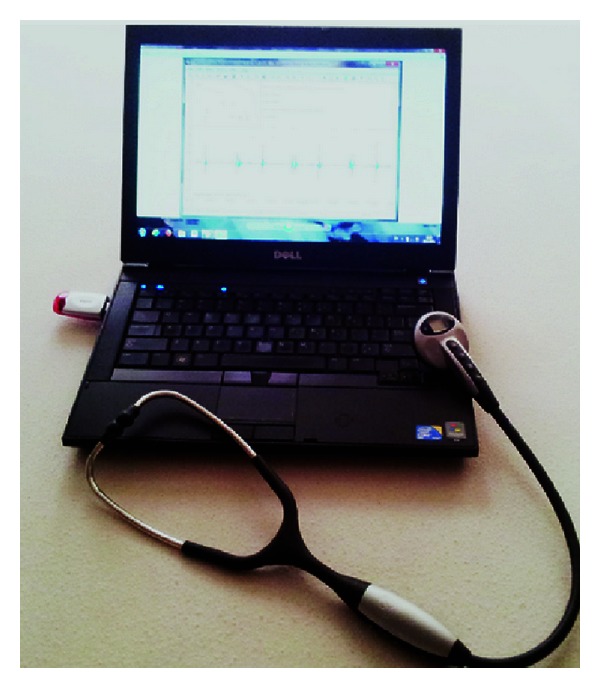
The equipment for acquisition of phonocardiograms.

Auscultation and phonocardiogram acquisition were performed at the Health Center “Zvezdara”, Belgrade, Serbia, using an electronic stethoscope (3M Littman 4100WS electronic stethoscope, Figure 1). All patients were in a sitting position during standard auscultative examination in the morning session (09–12 h AM). Data acquisition starts when a physician determines that the child is calm. During the recording of a PCG, patients did not perform any physical activity (or intellectual activity such as reading). Phonocardiogram acquisition was made by an expert in the standard cardiology protocol. Low-quality recordings (due to patient movement or similar reasons) have been rejected as nonrelevant. Only healthy children and children with PMVs were selected for generating the dataset used in this paper. Besides the PMV, children with other cardiac diseases like acute infection, anemia, or tachycardia are excluded from the experiment. Such recordings are not a part of the analyzed dataset.

The recordings were carefully examined by the pediatric cardiology expert. An additional examination (echocardiographic confirmation) was made at the University Children Hospital, Belgrade, Serbia, for the patients in the test dataset (97 recordings). The test PCG dataset was further divided into two groups:

- healthy group (children with healthy hearts) and
- PMV group (children with PMVs).

Echocardiography was used in order to confirm content validity of the groups. There are 49 children in Healthy and 48 children in PMV group.

Each phonocardiographic recording lasts for 8 seconds. PCGs were recorded with a sampling frequency of 8 kHz, with 16 bit amplitude resolution. The recordings were converted to wav format and downsampled to 1 kHz for further analysis.

## 3. Background

### 3.1. Prolapsed Mitral Valve (PMV) or Mitral Valve Prolapse (MVP) Is Also Known as Systolic Click Syndrome

It is a common valvular disorder and is often benign. However, it is also assumed as a disease which may cause serious cardiac disorders [5, 6]. There is no accordance regarding the prevalence of MVP: as reported in [5] the occurrence of MVP ranges from 5% to 15% and even up to 35%, while in [6] MVP is around 2-3%.

Various symptoms, including electrocardiographic (ECG) repolarisation, have been associated with PMV. A physician is most likely to use his stethoscope for initial PMV detection by auscultation (and phonocardiography). Auscultation still represents an important tool in pediatric cardiology. The misperception of a wide range of nonspecific symptoms led to the practice of acquiring screening echocardiograms from patients [6].

**Figure 2:**
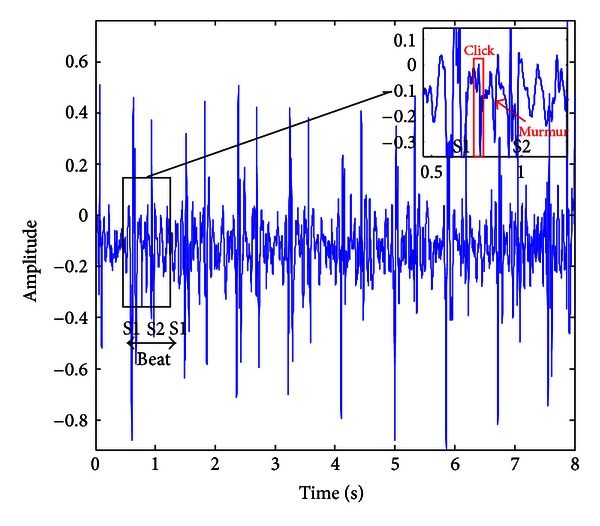
Phonocardiogram that corresponds to a child with prolapsed mitral valve (PMV).

In phonocardiography, PMV is usually manifested via a midsystolic click, as an isolated cardiac event or just before the systolic murmur (heart noise) appearance. The click syndrome is sometimes difficult to notice in the systole, and its detection depends on the skills of the physician [7]. Systole is defined as an interval between two fundamental heart sounds, S1 and S2 (i.e., *tic-tac*), visualized in a quasiperiodic phonocardiogram. An example of a PCG signal recorded on a patient with PMV is shown in Figure 2, where large amplitudes correspond to S1 and S2 sounds. S1 sounds are found after an electrocardiogram's R waves. Fluctuation in between such large peaks (heart beats) does appear to be interesting. A click as a singularity may be found difficult to follow in intervals between S1s and S2s (i.e., within systoles). This click existence is not always apparent to an average eye/ear.

There is a possibility of fault prognosis provided by phonocardiography, leading to misleading conclusions whether the signal corresponds to a healthy patient or a patient with PMV. Searching for specific cardiac events and features associated with PMV diagnosis in PCGs is an error-prone task. Such cardiac events within PCGs are crucial, indicating that findings may not be benign.

In early research of PMV treatment, in the 1970s, echocardiography was seen as an excellent method for diagnosing PMV. Echocardiography as a specific, noninvasive technique, can provide visualization of both mitral valve leaflets [8, 9]. Clinical features of the MVP, which can be associated with the click (click and murmur), as previously noted, are related with echocardiographically documented findings [10]. Bearing in mind the availability of appropriate equipment, the reliability of PMV detection is expected to increase. This paper deals with the global difference between a Healthy group and a PMV group of PCG recordings, due to the fact that the difference between these two groups is not often obvious.

### 3.2. Multifractals in Medical Signal Analysis

Self-similarity behaviour is an interesting property, found in many natural objects and phenomena. For instance, the structure of river networks, a cloud, the nervous system of humans, the structure of a tree, cauliflower, and so forth, have self-similar property: by observing such structures in different scales (almost) the same shape arises. Such objects (structures) are known as *fractals*, because they can be characterized by noninteger (fractional) dimension *D*_*F*_ [11]. The fractal dimension numerically describes how the irregular structure of objects and/or phenomena is replicated in an iterative way from small to large scales or vice versa and can be used for objective comparison and/or classification of different complex structures. The fractal dimension *D*_*F*_ can be estimated in different ways. One simple but efficient method is known as the *box-counting* method. This method involves the use of *n*-dimensional grid of nonoverlapping boxes of the side length **ε** within the space occupied by observed structure and counts the number of boxes, *N*(*ε*), covering the structure. The grid dimension equals *n* = *E* + 1, where *E* is an appropriate Euclidean dimension: *E* = 1, 2 or 3, for line, surface, or volume, respectively. If boxes of recursively different sizes are used for covering fractal object, the limiting value of *N*(*ε*), when *ε* tends to zero, follows the power law:

(1) $$\begin{array}{c} N\left(\varepsilon \right)\sim \varepsilon^{D_{F}}, \end{array}$$

that establishes the fractal dimension to be estimated [11, 12]. For artificially generated fractals, generated by using some predetermined rules, fractal dimension is scale invariant, that is, it has the same value regardless of the observation scale. Such objects are known as *monofractals* [12, 13]. Conversely, natural fractals are characterized by fractal dimension which varies depending on the scale. Such objects are referred to as *multifractals*.

Multifractals are derived as an extension of fractals that is appropriate for situations where a unique dimension is not enough. They were introduced by Mandelbrot in the 1980s for the purpose of measuring turbulent flow velocity [11]. In analysis of the regularity of a flow with velocity *v*, irregularities are concluded to be found. These events such as rapid singularity changes occur in different instants (from the Lebesgue measure in R^3^ space viewpoint). Hölder exponent *h*(*x*_0_) is assigned to each signal point *x*_0_. Therefore, each exponent value, *h*, corresponds to an appropriate set of points, *S*_*h*_. Multifractality can be seen as a wide range of Hölder exponents. Mapping, *h* → *D*_*h*_, defines a multifractal spectrum of a signal, in a way that for each fixed value *h* (set of points *S*_*h*_), the Hausdorff dimension *D*_*h*_ is calculated. Hölder exponent, *h*, is a measure of irregularity of a function *g* at observed point (a local feature). There exists a polynomial of order *n*, *P*_*n*_(*x*), and such a constant *K*, so

(2) $$\begin{array}{c} \left|g\left(x\right)-P_{n}\left(x-x_{0}\right)\right|\leq K{\left|x-x_{0}\right|}^{h} \end{array}$$

stands for all the points *x* in the neighborhood of *x*_0_. The spectrum is calculated using multifractal formalism:

(3) $$\begin{array}{c} D_{h}=\underset{q}{\inf}\left(q\cdot h-\tau \left(q\right)+k\right), \end{array}$$

where *q* is a real parameter used to describe the singularity of structure and determines the multifractal dimensions *D*_*h*_, *τ*(*q*) is a nonlinear function, and *k* is a constant. For *q* > 1 strongly singular structures are emphasized, for *q* < 1 less singular structures are emphasized, while for *q* = 1, *D*_*h*_ equals information dimension. 

There is yet another approach for introduction of multifractality. Fractal dimension, *f*_*h*_, can be defined for a set of Hölder exponents of points that are within the range [*h*, *h* + Δ*h*]. Such set may be considered monofractal. Legendre transform can be used for the relation between function *τ*(*q*) and a multifractal dimension, *f*_*h*_:

(4) $$\begin{array}{c} \tau \left(q\right)=q\cdot h\left(q\right)-f_{h},\;h\left(q\right)≅\alpha \left(q\right)=\frac{d\tau \left(q\right)}{dq}. \end{array}$$

Parameter *α* represents an approximation of the Hölder exponent, *h*, where maximum of spectrum (*h*, *f*_*h*_) corresponds to the Hausdorff dimension, *D*_*h*_ [13]. In order to point out the multifractal formalism and thus to explain the alpha (*α*) as an approximation of Hölder exponent, we have introduced the expression (4).

In general, multifractality is verified in the literature as effective concept in analysis of medical signals and images [13, 15, 16]. Legendre singularity spectrum can be calculated for different values of**α**and shows a global aspect of the content in a medical signal. It results in a smooth concave (decaying) function of Hölder exponent, *f*(**α**), that gives a general information about behavior of the analyzed set of points. A simple and efficient method for estimating the multifractal spectrum is the histogram method (and it is based on the box-counting method) [16].

**Figure 3:**
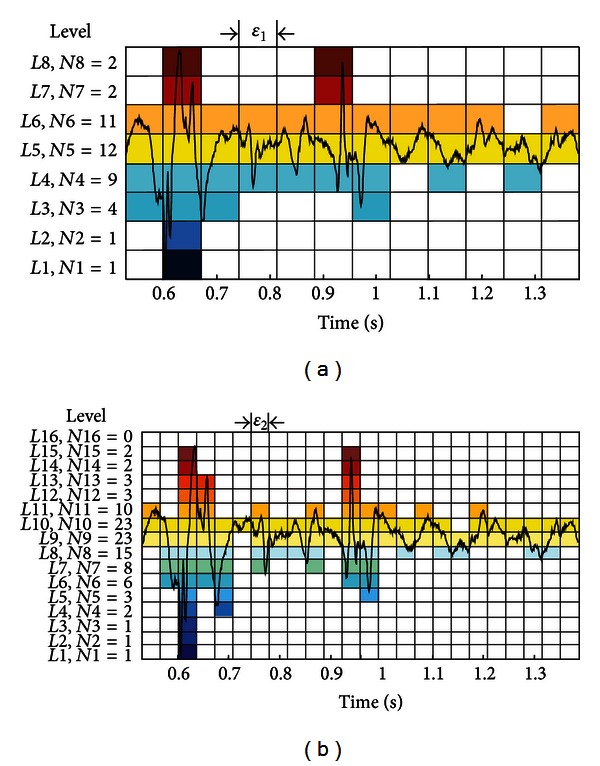
An illustration of the box-counting technique as a basis for the histogram method: (a) *ε*_1_ = 1/12, (b) *ε*_2_ = 1/24.

Figure 3 gives an illustration of the box-counting technique as a basis for the histogram method. It is applied to a given structure *S* (e.g., to a part of the PMV record, Figure 2), which is then divided into the nonoverlapping boxes *B*_*i*_ of size *ε* so that *S* = ∪_*i*_ *B*_*i*_. Each box *B*_*i*_ is characterized by measure *μ*(*B*_*i*_) (here the signal level is used as a measure *μ*, Figure 3(a)). The coarse Hölder exponent of the subset *B*_*i*_, corresponding to given measure *μ*, is calculated as

(5) $$\begin{array}{c} \alpha_{i}=\frac{\ln\left(\mu \left(B_{i}\right)\right)}{\ln\left(\varepsilon \right)}. \end{array}$$

By changing the box size, Figure 3(b), the value of the coarse Hölder exponent changes as well. In the limiting procedure, as box size tends to zero, Hölder exponent **α** at a specific location, within the observed signal (structure), becomes more precise. The Hölder exponent describes the local regularity of structure *S*. Finally, the distribution of **α** exponents, *f*(**α**), that is, the multifractal spectrum (Legendre spectrum) is determined. The multifractal spectrum describes the global regularity of observed structure. In this paper, the estimation of Legendre spectrum (based on the box-counting technique) is performed using FracLab software [36].

## 4. Healthy versus PMV Phonocardiograms-Multifractal Explanation

### 4.1. Initial Examination

We considered 97 PCG records that belong either to healthy patients or patients with PMV, where diagnoses are confirmed by the analysis of phonocardiograms and echocardiograms (echos). There are 49 recordings from 49 healthy children and 48 recordings from 48 children with PMV.

**Figure 4:**
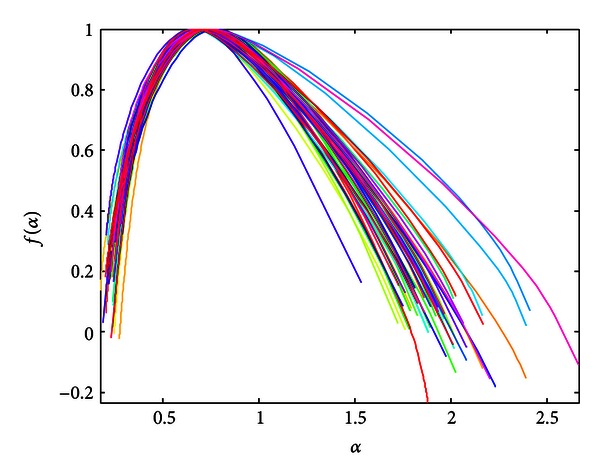
Legendre multifractal spectra calculated for healthy patients (findings confirmed by echo analysis).

In Figure 4, we show curves of multifractal spectra calculated for healthy children (49). One can notice the multifractality of the PCGs.

**Figure 5:**
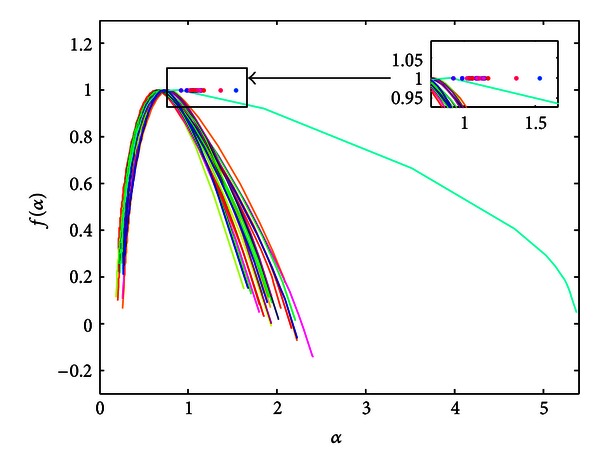
Calculated Legendre multifractal spectra for patients with PMV (findings confirmed by echo analysis).

Multifractality is also evident in the group of children with PMVs. In order to compare the spectra between the groups, Legendre multifractal spectra for PMV recordings are calculated under the same conditions as spectra of the healthy patients. A certain number of PMV recordings were displayed as points (as “monofractals,” Figure 5). Spectra calculation under the same conditions is based on the use of predefined parameters which are calculated for healthy patients. The parameters are related to alpha values corresponding to healthy recordings, calculated by [36]. Multifractal spectrum curve, (*α*, *f*(*α*)), could not be displayed for a certain number of PMV signals. This implies that previously calculated set of values determined for the healthy patients do not correspond to the abovementioned PMV recordings.

**Figure 6:**
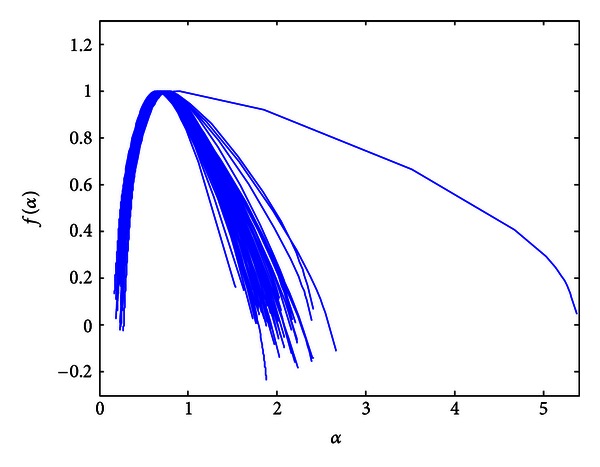
Multifractal spectra of the PCG dataset (excluding spectra of signals displayed as points).

Since the spectra are calculated under the same conditions that correspond to healthy recordings, the fact that only PMV signals were displayed as points is used in the classification algorithm. Each phonocardiogram that is displayed as a point is automatically classified as a signal that belongs to a patient with PMV. The rest of PMV signals (24 recordings) were displayed as curves in the multifractal domain. Our goal is to further examine their multifractal spectra in order to make a distinction between them and Healthy group. The 73 signals from both groups are shown in Figure 6, where they are presented by multifractal curves. The similarity between the spectra in Figure 6 is expected since the distinction is difficult to make by eye/ear.

#### 4.1.1. Feature Analysis—Healthy versus PMV Classification

**Figure 7:**
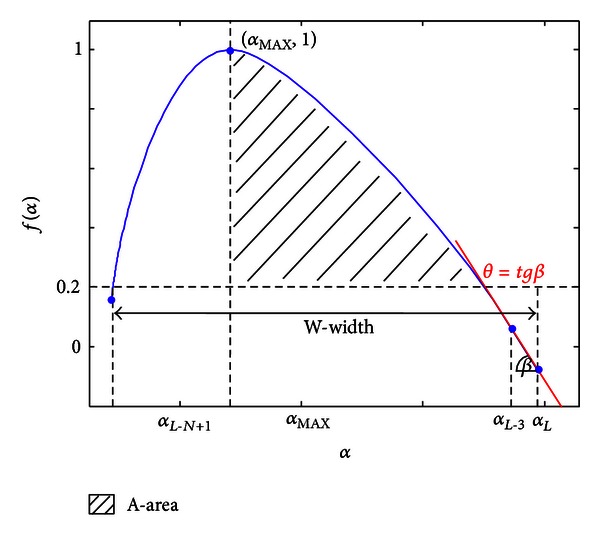
An example of a multifractal spectrum curve with analyzed parameters.

Obtained multifractal spectra curves *C*_*j*_, (*j* = 1,…, 73) are sets of points (*α*(*n*_*j*_), *f*(*α*(*n*_*j*_))), *n*_*j*_ = *L*_*j*_ − *N*_*j*_ + 1, …, *L*_*j*_. We examined several features of such sets: shapes of the curves for Healthy and PMV group (width of a curve and area under a curve), location of maxima, characteristics of a curve shape for large alpha values, and so forth.  Figure 7 shows an example of a multifractal spectrum curve with tested characteristics: width (*W*), area (*A*), alpha value of the maxima (*α*_MAX_), and slope of the curve (*θ*).

**Figure 8:**
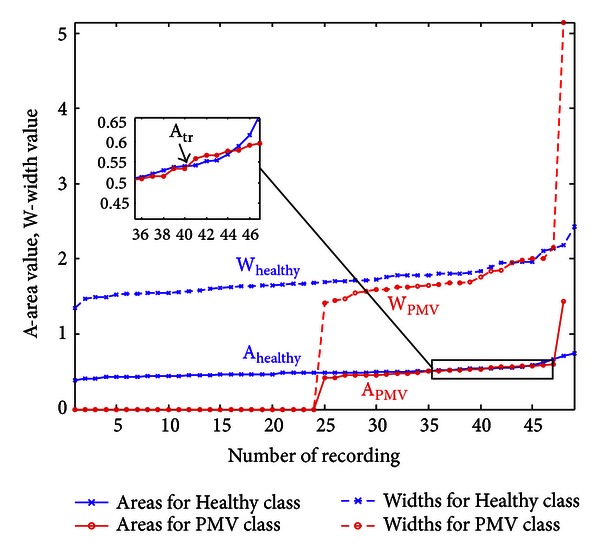
Widths and areas for Healthy and PMV group, respectively, presented in increasing order.

Width of a spectrum is calculated as a difference between alpha values in ending points of the curve as *W* = *α*(*L*) − *α*(*L* − *N* + 1). Area *A* is calculated among lines *f*(*α*) = 0.2, *α* = *α*_MAX_ and a multifractal spectrum curve. If a signal spectrum is displayed as a single point, area *A* and width *W* will be zero.  Figure 8 shows calculated *A* and *W* values for 97 patients. The relevance of these features for threshold settings may be seen in their sorted presentation. By visual inspection, we notice a range of areas (and widths) where most of the signals (both normal and PMV) concentrate.

In order to compare the similar multifractal spectra, in the proposed approach we move the maxima of all the *f*(*α*) curves to the point (*MaxPos*, 1). The location of this point, *MaxPos*, is calculated as an average of all maxima locations, **α**_MAX_. This gives a better insight into the shape characteristics of the curves. For the comparison of the multifractal curves, the Legendre multifractal spectrum with maximum value 1 is calculated for every signal.

**Figure 9:**
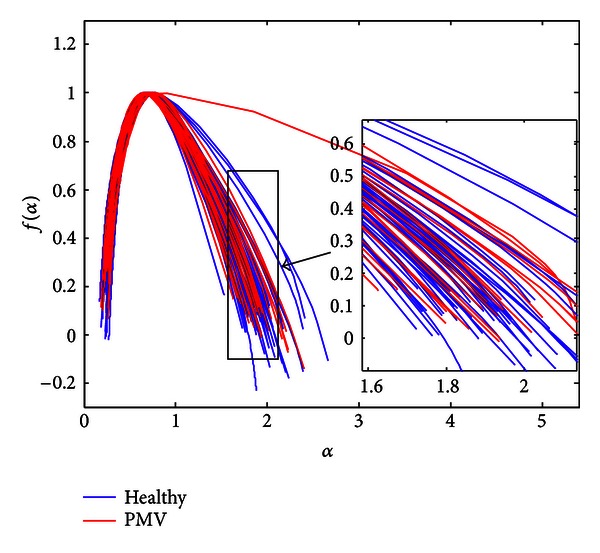
Slopes of the curves in the right side of spectra for Healthy and PMV group.

In the analysis of multifractal spectra, we noticed that relative changes of curve shapes on their right sides may carry valuable clinical information in accordance with the multifractal theory. Actually, we noticed that the slope angles as features of the curves may be relevant (Figure 9). The slope parameter for a curve *C* is calculated as

(6) $$\begin{array}{c} \theta =\frac{\left|f\left(\alpha \left(L\right)\right)-f\left(\alpha \left(L-3\right)\right)\right|}{\left(\alpha \left(L\right)-\alpha \left(L-3\right)\right)}. \end{array}$$

The parameter *θ* is assumed to be significant, and it is used in the classification procedure. When multifractal spectrum of a signal is displayed as a point, the slope parameter has zero value.

### 4.2. Proposed Algorithm

We propose a two-step algorithm for distinguishing healthy records from PMV ones, based on the Legendre multifractal spectra. According to the feature analysis of the curves, we noticed that the slope parameter may be decisive. We also noticed that most of the spectra fall within a narrow range of area values.

For obtained multifractal spectra curves, we apply a two-step algorithm:

1. dividing spectra into two sets based on area feature, *A*, and
2. differentiation in two classes (Healthy and PMV) based on the slope parameters calculated for both of the sets (obtained according to area values in the previous step).

**Figure 10:**
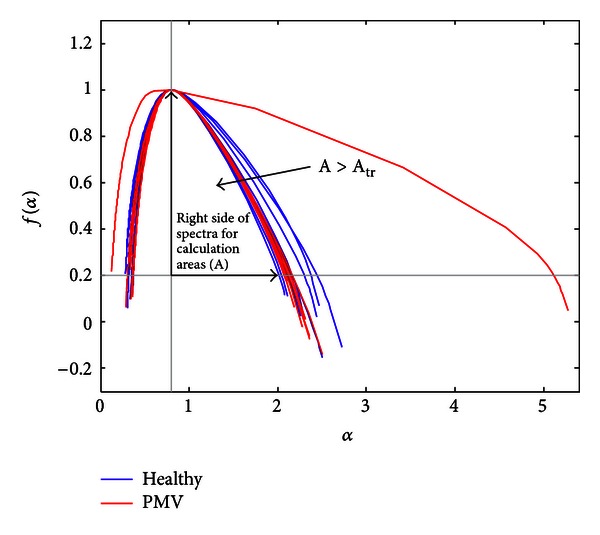
The large-area spectra selection.

**Figure 11:**
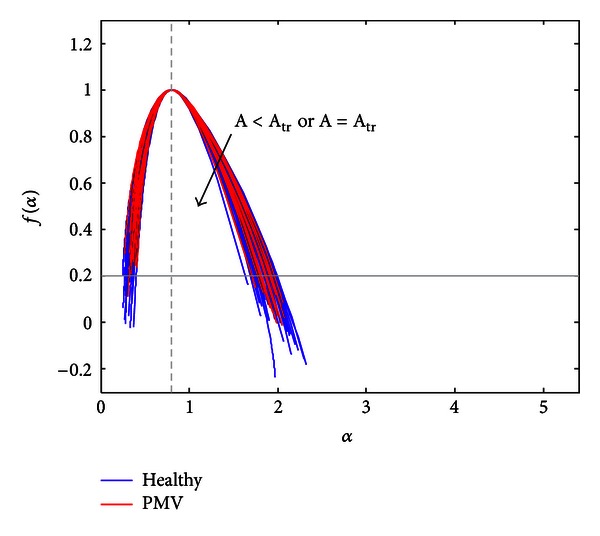
Spectra selected for *A* ≤ *A*_tr⁡_.

The first step in the classification is realized using the threshold *A*_tr⁡_, for dividing curves into two sets. For *A* > *A*_tr⁡_, curves that have large area values are selected. They can possibly be considered as outliers when displaying the spectra. Most of the curves are selected for *A* ≤ *A*_tr⁡_. We arbitrarily chose the first intersection of area trends in Figure 7 for the threshold value *A*_tr⁡_ (0.55). The curves selected for *A* > *A*_tr⁡_ and *A* ≤ *A*_tr⁡_ are represented in Figures 10 and 11, respectively.

**Figure 12:**
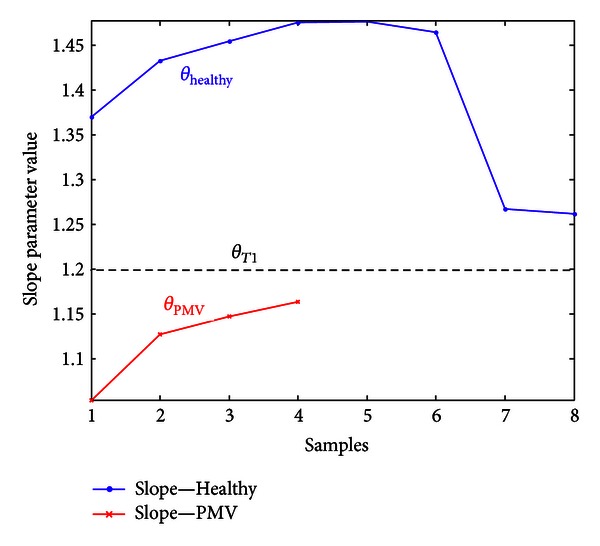
The slope parameter differentiation for large-area spectra (*A* > *A*_tr⁡_).

In the second step of the algorithm, parameter *θ* is calculated for curves from both sets (shown in Figures 10 and 11). If *A* > *A*_tr⁡_, the slope *θ* is compared with predefined slope threshold *θ*_*T*1_. If slope value *θ* is less than the threshold value,

(7) $$\begin{array}{c} \theta <\theta_{T1}, \end{array}$$

recording is automatically classified as a recording with a click syndrome (PMV class). This is shown in Figure 12. 

If *A* ≤ *A*_tr⁡_, the slope parameter *θ* is compared with the predefined threshold *θ*_*T*2_. If slope parameter *θ* is less than the threshold,

(8) $$\begin{array}{c} \theta <\theta_{T2}, \end{array}$$

recording is classified as PMV. Otherwise, it is classified as an element of the Healthy class. In the case of *A* ≤ *A*_tr⁡_, by visual inspection, we also noticed that most of the curves have *α*_MAX_ values lower than the *MaxPos* value. Spectra displayed as points have higher values (*α*_MAX_) than the *MaxPos*. In the proposed algorithm, *α*_MAX_ values (and *MaxPos*) are not used for the classification. The relevance of *α*_MAX_ parameter is still questionable.

**Figure 13:**
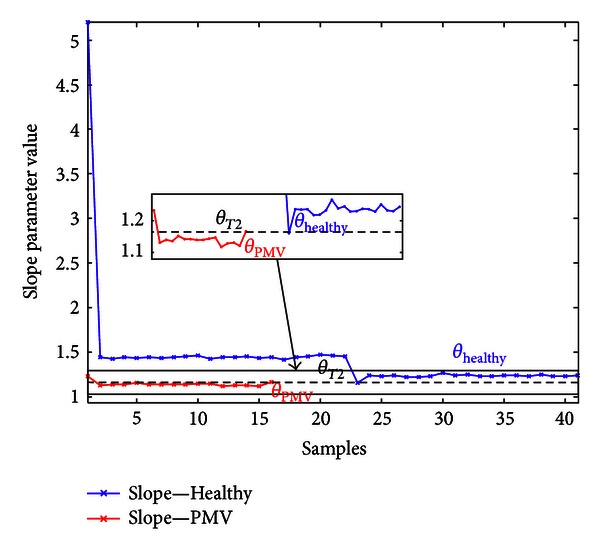
The values of slope parameter for Healthy and PMV class for *A* ≤ *A*_tr⁡_.

All threshold values (*A*_tr⁡_, *θ*_*T*1_, *θ*_*T*2_) are chosen empirically, by observing the spectra with respect to particular tolerance (offset) when determining the value. Empirical lines of separation are set by examination. Figures 12 and 13 represent calculated slope parameters for the set of curves with large area values (*A* > *A*_tr⁡_) and small area values (*A* ≤ *A*_tr⁡_), respectively. In Figure 12, twelve values of slope parameters are showed for 12 curves (*A* > *A*_tr⁡_), where four among them belong to PMV class. 

The slope parameter is relevant for the other group, as well. In Figure 13, the rest of PCG signals (61 recordings) are presented via slope values. We can freely add 24 PCG recordings to this group of signals, since they were not manifested as multifractal curves (area *A* and slope parameter *θ* have zero value; threshold *θ*_*T*2_ is real positive constant).

## 5. Simulation Results 

### 5.1. Results of the Proposed Algorithm

In the set of 97 PCG recordings, we achieved an accuracy of 96.91%. Obtained results of the simulation are shown in Table 1.

**Figure 14:**
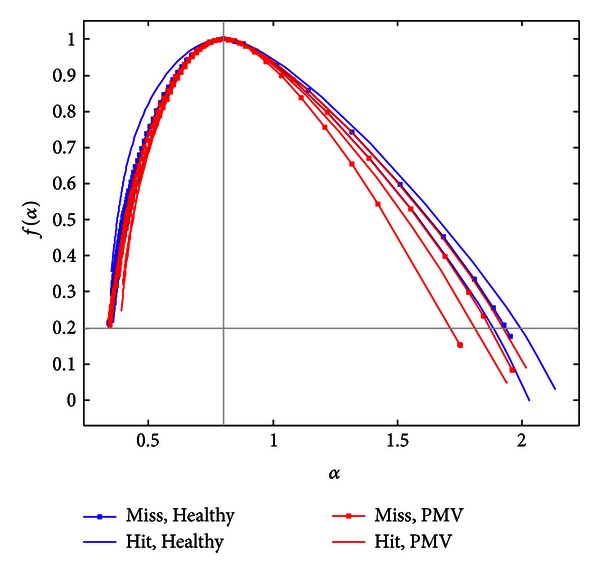
Examples of correctly classified spectra and misclassified spectra.

Even in the case of overlapping of the spectra curves, the algorithm gives excellent results in differentiating PMV recordings from healthy recordings. In Figure 14, three misclassified spectra are shown, and two correctly classified spectra for each group. For the purpose of presentation, we keep colors for signal differentiation (blue—for Healthy and red—for PMV recordings).

In the validation study, we analyzed additional 20 PCG recordings that were not used for setting the thresholds (naive dataset). Totally, 20 children (*M* = 9, *F* = 11) contributed to the realization of this dataset (one patient - one signal). Their class (healthy or PMV) affiliation was not known to the authors during testing the dataset. The results in validation study will be explained in the following subsection.

### 5.2. Echocardiographic Confirmation and Validation Results

The examination of recorded PCG dataset is followed by echocardiographic examination at the University Children Hospital in Belgrade. It is confirmed which of 117 recorded phonocardiograms belong to which (Healthy or PMV) class. Our analysis is performed only on these groups. In standard phonocardiographic analysis, cardiology experts may have uncertainties among such dataset which are resolved with the echocardiographic study.

As previously mentioned, we tested 20 additional PCG signals provided by the physician, using the proposed algorithm (test) with the fixed thresholds. Our algorithm gave satisfying results in comparison to the echocardiographic study. The validation results are presented in Table 2. All 10 PMV recordings were predicted correctly as PMV. Two out of 10 healthy recordings were misclassified. 

The proposed procedure is shown to be efficient and simple. Even though the box-counting technique is sometimes neglected because of masking the singularities, the proposed algorithm has overcome this limitation. 

The multifractal spectra calculation is also simpler for realization than wavelet-based methods. Particular anomaly is detected using characteristic features of the multifractal spectra curves. Figure 9 points out the part where parameter *α* is large. The click syndrome is expected to have an effect on the right side of the spectra unlike the recordings from the healthy group, where such clicks have not been found (or where the particular behaviour in the systole is not a long-term event).

Even though the whole recording was used, large amplitudes in time domain that correspond to S1s (and S2s) are not relevant in the proposed classification procedure, which is based on multifractal analysis. The existence of nonregular cardiac events (possible clicks) affects the right side of multifractal spectra curves as opposed to the regular heart sounds (S1s and S2s). Time localization of the clicks was not a part of this analysis.

This and similar approaches can indicate signal irregularities to users of electronic stethoscopes without large experience or skills in auscultation and phonocardiography. In this way, the misinterpretation of phonocardiograms may be significantly decreased.

## 6. Conclusion

We have been primarily focused on the criteria for obtaining the clear distinction between patients with diagnosed PMV and healthy patients using phonocardiography. We have tested 97 PCG recordings, where each record is 8 seconds long, with downsampled frequency of 1 kHz. Echocardiographic examination confirmed clinical findings in PCGs. proposed algorithm achieves high accuracy (96.91%). In the evaluation step of the study, we used additional 20 PCG signals from another set of patients (20 children). Only two signals were misclassified according to the echocardiographic examination.

At this point, the research about automatization of the thresholds has not been finished. Nevertheless, there are clear indications about the direction of future research, especially with respect to robustness. We want to emphasize that each record is obtained from a different patient and gives a representation of a different “pumping machine” (heart) function. Therefore, the features, thresholds, and generally the algorithm can be considered robust for a large population. An increase of the training dataset may set thresholds more accurately.

Further research will be primarily oriented towards further evaluation. This involves the use of additional classes of signals (signals belonging neither to Healthy nor PMV group). We believe that applying well-known signal processing techniques may contribute to the development of a cost-effective computer-aided diagnosis system based on phonocardiograms. Decreasing the use of complex equipment for screening by efficient low-cost approaches may be of great importance. It is necessary to have large datasets of phonocardiograms for this purpose with confirmed diagnosis and labeled cardiac events.

## Figures and Tables

**Table 1:** Simulation results.

Class	Number of recordings (with echo confirmation)	Number of hits (the proposed PCG-based algorithm)	Accuracy
Healthy	49	48	97.96%
PMV	48	46	95.83%

Total	97	94	**96.91%**

**Table 2:** Validation results.

Class	Number of recordings (with echo confirmation)	Number of hits (the proposed PCG-based algorithm)
Healthy	10	8
PMV	10	10

Total	20	18
